# Living with a long-term condition: Understanding well-being for individuals with thrombophilia or asthma

**DOI:** 10.3402/qhw.v11.31530

**Published:** 2016-08-16

**Authors:** Jennifer K. Roddis, Immy Holloway, Carol Bond, Kathleen T. Galvin

**Affiliations:** 1 Faculty of Health and Social Sciences at Bournemouth University, Bournemouth, UK; 2 College of Life, Health and Physical Sciences, University of Brighton, UK

**Keywords:** Long-term condition, asthma, thrombophilia, living with, gaining knowledge, patient perspective, qualitative, constructivist grounded theory

## Abstract

A range of literature has explored the experience of living with a long-term condition (LTC), and frequently treats such experiences and conditions as problematic. In contrast, other research has demonstrated that it may be possible to adapt and achieve well-being, even when living with such a condition. This tends to focus on meaning and the qualitative experience of living with an LTC, and offers alternative perspectives, often of the same or similar conditions. As a result of these conflicting views, this study chose to consider two conditions which, though they may lead to life-threatening illness on occasion, do not appear to impact significantly the lives of all those affected on a daily basis. The aim of this research was to explore and explain how people make sense of two long-term, potentially life-threatening health conditions, namely, thrombophilia and asthma. In doing so, it specifically considered the contribution made by information about the condition. A constructivist grounded theory approach was adopted; this enabled the generation of a theory regarding how people make sense of their LTC, whilst acknowledging the social circumstances in which this was situated. Semi-structured interviews were conducted with 16 participants who had given consent to take part in the research. The findings demonstrate that participants undergo a two-stage process—*gaining knowledge* and *living with a long-term condition*. The theory based on these findings indicates that those who are knowledgeable about their condition, making informed decisions in relation to it, and accept their condition are able to live with it, whilst those who do not accept their condition do not fully adapt to it or integrate it into their lives.

Approximately 20 million people in England have at least a single long-term condition (LTC). The definition of LTC adopted for the purpose of this study is that offered by the Department of Health in England, which states that an LTC is “one that cannot currently be cured but can be controlled with the use of medication and/or other therapies” (Department of Health, 2010, p. 4). People with such conditions account for 70% of healthcare spend (Parliament, 2013), more than 70% of inpatient bed days, 65% of outpatient appointments, and 50% of general practice appointments (Royal College of General Practitioners, 2011). Thus, the extent and impact of LTCs is wide-ranging, both for society as a whole and for affected individuals.

The literature describes how having an LTC negatively impacts the self, quality of life, and ability to carry out day-to-day activities. Charmaz (1999) describes how suffering caused by chronic illness often results in a changed self. The findings of qualitative research considering experiences of people affected by chronic obstructive pulmonary disease indicate themes of struggling, fear, blame, fatalism, and hidden disability (Clancy, Hallet, & Caress, 2009). Kurpas et al. (2013) confirm that the level of illness acceptance for people with chronic respiratory illnesses is low. Individuals with angina, arthritis, asthma, and diabetes experience higher rates of depression than those unaffected by these conditions (Moussavi et al., 2007). Individuals with rheumatoid arthritis and asthma experience life as problematic; the body does not function as it should; illness-related disabilities provide a strong focus, and those affected perceive themselves to be socially inadequate, for example, being unable to continue in one's profession (Price, 1996). A review of outcome measures used in relation to rheumatoid arthritis shows that those with the condition score lower in quality of life measures when compared to those without, to the extent that some patients obtained scores which showed that they were in “states worse than death” (Kingsley, Scott, & Scott, 2011, p. 597).

In direct contrast, the findings of qualitative research carried out by Sanderson, Morris, Calnan, Richards, and Hewlett (2010) and Graves, Scott, Lempp, and Weinman (2009) show that people with rheumatoid arthritis are able to feel well even when disabled by the condition. Women affected by urinary incontinence attempt to regain power over the condition and strive to live a normal life (Hägglund & Ahlström, 2007). Acceptance of one's condition is possible for those with diabetes, chronic fatigue, and inflammatory bowel disease (Cooper, Collier, James, & Hawkey, 2010; Kenealy, Kyle, & Simmons, 2008; Wilson, Whitehead, & Burrell, 2011), and is a contributory factor in well-being (Berntsson, Berg, Brydolf, & Hellström, 2007; Hammarström & Torres, 2012). Galvin and Todres (2011) have also pointed to a new perspective on well-being that includes finding possibility within illness that is relevant to these understandings.

The literature demonstrates that an LTC may be perceived in different ways. It can be considered acceptable and something that can be lived with by those affected, or seen as something to be contended with. The conditions examined in this study were those which might be perceived as threatening or problematic by those affected, as they can lead to acute illness on occasion.

The literature detailed above offers some insights into how those with an LTC perceive life with it; these may be transferable to those who have thrombophilia or asthma. This is of particular use as there is a lack of research concerning the views of those living with thrombophilia in particular.

The literature on chronic pain, in contrast to the conditions discussed herein, demonstrates its tendency to be relentless, affecting the individual on a daily basis (McHugh & Thoms, 2001). At least some of those who experience chronic pain report anxiety (Crowe et al., 2010; McCracken, Spertus, Janeck, Sinclair, & Wetzel, 1999; Osborn & Rodham, 2010), and many report a body which is separated from the mind (Afrell, Biguet, & Rudebeck, 2007; Crowe et al., 2010; Osborn & Smith, 2006; Paulson, Danielson, & Söderberg, 2002). These are contrasted with the theoretical concepts identified in the current study.

This study offers theoretical insights into the experience of living with an LTC, and how individuals make sense of this. Whilst it uses the examples of thrombophilia and asthma, it develops concepts which may be applied to other LTCs (with the exception of chronic pain) and how those with such conditions integrate them into their lives.

## Methods

The aim of this study was to explore and explain how people make sense of two long-term, potentially life-threatening health conditions, namely, thrombophilia, and asthma. It did so by investigating where and how individuals obtained information and its contribution to and usefulness for understanding their condition, by illuminating beliefs and understanding of information about their condition, and examining any other factors which may affect the process experienced by those living with thrombophilia or asthma.

A constructivist grounded theory approach was adopted, enabling the production of a theory regarding how people make sense of their LTC. This type of grounded theory contends that knowledge of and meaning in the world is not discovered, but instead constructed and reconstructed by individuals and/or society (Charmaz, 2003). Constructivist grounded theory also stresses the importance of meaning, process, and interaction and uncovers how people define and construct their social reality within the social context. This assists the researcher to construct and interpret the participants’ worlds (Charmaz, 2014).

The perspectives of people with thrombophilia were initially chosen as, unlike many LTCs considered in the literature, this condition did not seem to change people's lives on a day-to-day basis, although it might lead to life-threatening illness on occasion. Following the tenets of grounded theory, theoretical sampling, where the researcher selects individuals for inclusion on the basis of their potential contribution to the further development and explication of the theoretical concepts emerging from the research (Charmaz, 2006), was adopted. This indicated that emerging concepts ought to be revisited with individuals affected by a second condition; asthma was chosen due to its similarity as a second condition, which may not change people's lives on a daily basis, but can lead to serious illness. Asthma may lead to serious exacerbations, which can in extreme cases be fatal. Thrombophilia can cause blood clots which, if not diagnosed and treated, can be life-threatening. In each case, treatment can be used to reduce the likelihood of serious illness but cannot prevent it entirely. Restrictions may result from either the medication or the condition itself. Those with thrombophilia will be advised not to take long-haul flights without some form of prophylactic medication, and those taking medication will be required to undergo frequent blood tests. People with asthma can be affected by weather and pollen conditions, suffer from allergies, and find strenuous activities to be tiring. Despite this, most individuals with thrombophilia or asthma are able to go about their day-to-day lives with little concern for their condition.

As is usual in qualitative research, an initial literature review was conducted at the beginning of the study in order to identify knowledge gaps and contextualize the research (Dunne, 2011). The identification of further relevant literature was guided by and used in dialogue with the findings.

Data collection and analysis were conducted iteratively (Glaser & Strauss, 1967) and, as stated, theoretical sampling adopted to follow up emergent concepts. This was carried out by accessing individuals initially through two local hospitals and a national charity working with people who have had clots. The concepts arising through this data source were then followed up through exploring the perspectives of people with another LTC, this being asthma. Saturation has been attempted although it is difficult to achieve (Charmaz, 2014). Data collection stopped when no specific ideas important for the emerging theory arose, and the concepts and categories relevant to the research aim had been explained.

Data were collected through semi-structured interviews (Kvale & Brinkman, 2009), which became increasingly structured during the research and progressively focussed on important concepts. As is usual in qualitative research, the questions changed over the course of the interviews, but questions asked initially included, for example, “Can you tell me about life with thrombophilia/asthma?”, “Where did you get information from?”, “Did you look for information?”, “How do you feel about having thrombophilia/asthma?”. Interviews were conducted face-to-face either in the participants’ homes or in their workplace, on university premises, or by telephone. Whilst the researcher was aware of the possibility that collecting data through different modes could lead to differences in the data collected, consideration confirmed that the different modes had little, if any, impact on the data collected.

Criteria for involving individuals in the study included those aged between 18 and 85, able to speak English, and affected by either thrombophilia or asthma. Individuals were recruited either through a local hospital (thrombophilia only), a national charity which supports those who have had blood clots (thrombophilia only), or a local advert (both thrombophilia and asthma). Recruitment took place between autumn 2008 and spring 2015, by the researcher or colleagues at a local hospital and a national charity. Table I provides details of the participants, together with their Unique Reference Number (URN), demonstrating that data were collected from people of both genders and with both conditions.

**Table I:** Participant details including reference number, gender, age range, condition, access to the Internet, and highest qualification.

Participant's Unique Reference Number (URN)	Male/female	Age	Internet access?	Highest qualification	Condition
P081	Female	51–65	No	None	Thrombophilia
P083	Male	36–50	Yes	GCSE	Thrombophilia
P091	Female	36–50	Yes	CSEs and GCEs	Thrombophilia
P093	Male	36–50	Yes	HND	Thrombophilia
P094	Male	51–65	Yes	O-levels/City and Guilds	Thrombophilia
P111	Female	51–65	Yes	Works for relevant charity	Thrombophilia
P112	Female	25–35	Yes	Degree	Thrombophilia
P113	Male	51–65	Yes	Degree	Thrombophilia
P121	Female	36–50	Yes	Degree	Thrombophilia
P143	Female	36–50	Yes	Masters	Thrombophilia
P131	Male	51–65	Yes	PhD	Asthma
P141	Female	25–35	Yes	Masters	Asthma
P142	Female	25–35	Yes	Degree/equiv	Asthma
P145	Male	36–50	Yes	Degree	Asthma
P146	Female	36–50	Yes	PhD	Asthma
P147	Male	36–50	Yes	PhD	Asthma

Ethical approval was acquired from the NHS Research Ethics Service (08/H0201/87) and from the university, and permission received from the charity. Participants gave written consent to take part in advance of the interview and also gave verbal consent at the beginning of each interview. Interviews were recorded and transcribed by the researcher, with all identifying features (names, places) removed. The main point, however, is that the researcher tried to act ethically throughout the study.

## Findings

The sample involved ten individuals with thrombophilia (four male and six female; aged 25–60 at the time of interview) and six with asthma (three male and three female; aged 32–51). Three participants were formally interviewed a second time.

A two-stage process was identified which details how people make sense of their LTC, incorporating how people learn about and understand thrombophilia, and, following further theoretical sampling, asthma. For the purposes of this paper, findings are presented in three parts. The findings in relation to those with thrombophilia are detailed first, followed by those relating to asthma, and finally theoretical insights into having an LTC are offered. The first stage is designated *gaining knowledge*.

### Gaining knowledge

The first, pre-diagnosis phase occurs for those individuals who become aware that they may have some sort of illness. This can result from the diagnosis of a family member, a healthcare professional suspecting a particular condition, or from the linking of symptoms by individuals who recognize that these may be related. Some people will experience their condition for some time prior to diagnosis. As a result, individuals will begin to form constructs about their condition.

Healthcare professionals had advised several participants that they might have thrombophilia. For example, one man was informed by his healthcare professional that thrombophilia may have been the cause of a deep vein thrombosis (DVT) he had experienced, saying “I'd never heard of it. I'd never heard of Factor V … suggested that I go back to see somebody with an expertise in that sort of thing” (URN P113).

Some people had been told by members of their family that thrombophilia existed in the family and that they could be affected. One participant was alerted to the possibility that she may have thrombophilia following her sister's diagnosis.

It started with my sister, erm she had a problem with her eye and they found out that she had a blood clot behind her eye erm and they did tests for several months because they couldn't work out what it was and then found out that she has the factor V … her doctor at the hospital had told her to tell us to get checked. (P091)

Diagnosis is the point at which individuals received confirmation of their condition, for most people through consultation with a healthcare professional. A number of people were not diagnosed as soon as they experienced symptoms, either due to a missed diagnosis or because the individuals delayed seeking medical advice.

A number of participants with asthma had been diagnosed at a young age and had lived with knowledge of it for most of their lives. They started to understand this when their parents were informed of both diagnosis and ways to manage the condition:

I think they diagnosed it pretty early and I think, it didn't get too drastic, sort of erm, a really bad point and they did diagnose it and to be honest with you I do remember having it from, from ever, I always remember having it. (P141)

These individuals were too young when diagnosed to see this as surprising. It led them to know asthma from a young age and much of the information they gained came from sources other than healthcare professionals.

For those with thrombophilia, in particular, diagnosis was not always a straightforward process. Delayed diagnosis can result from illness prior to the development of appropriate tests, results not being communicated to individuals, or when healthcare professionals do not recognize the cause of symptoms. Those who went undiagnosed experienced further illness before a diagnosis was received and they were provided with appropriate information and advice about the condition. For example, one woman had suffered two clots and received a negative test result.

At the time the thrombophilia tests then were really, I mean this is 30 years ago, they were really not very good at all and they at the time it came back and they just looked for things like lupus and stuff like that and it all came back negative so they said no it can't be anything wrong, it's just bad luck. (P111)

Of those who encountered a delay in diagnosis, most had previously experienced the condition. They were aware of symptoms, had been ill, and, as a result, had gained extensive tentative information before receiving a diagnosis. These experiences thus contributed to individuals’ knowledge, despite often occurring before their condition had been given a name.

Subsequent to diagnosis, individuals began to use their knowledge to determine how to deal with and manage the condition on a day-to-day basis so that they experienced well-being. They also amalgamated existing, relevant information with this ongoing knowledge. Information and understanding was usually gained from a number of sources, including healthcare professionals, family and friends, the Internet, books and other media, and, most significantly, experience.

One individual had discussed his hereditary thrombophilia with his mother

My mum's sort of like she had her two penn'orth to add when I was thinking about taking the heparin injection and it's probably the first time she's really said about it to me, apart from the fact she's oh I I don't take ‘em and I've gone through my whole life not knowing you know that I've had it. (P093)

whilst another had gained information about asthma from friends, saying “There was people at school and stuff that had the asthma, asthma inhalers and, you know, it was, you, you know, I knew quite a lot about it … speaking to some peers and so on” (P145).

Experiential knowledge formed a significant aspect of the information held by participants.

This included information about illness triggers, symptoms, treatment options, the outcome of not taking treatment as prescribed, the impact of illness on their relationships with healthcare professionals, the long-term effects, and day-to-day experiences of having and managing their condition. This knowledge held more meaning as it was personal to the individual; they found out how the condition affected them personally rather than merely accepting generic information provided by a healthcare professional. One individual said of her asthma inhalers:

I don't use my brown inhaler enough because when I'm not ill, say for example over Christmas I had a bit of a cough and a bit of a cold about me so I used my brown inhaler regularly and I was very conscious to take it, but then when I'm feeling fine, I don't really you know, I'll go weeks without taking my blue inhaler and forgetting to take my brown inhaler. And then you know it's only when something triggers it then I'm, oh, quick, have I got the blue inhaler. But then it's silly 'cause I know that taking the brown inhaler regularly really helps, but I do find when I first take it it feels worse … I feel quite tight chested at first. (P141)

Despite having experienced a pulmonary embolism (PE), one woman with thrombophilia had learned to recognize that day-to-day life with thrombophilia did not necessarily mean she would die of a blood clot.

Not every pain in my leg is is a clot and I've learnt to live with that. Not every pain in my chest is a clot, not every time I have a cough or I feel a bit breathless am I about to die from a pulmonary embolism. (P111)

Thus, individuals will gain understanding of their condition from a range of information sources. The Internet can be useful or considered to be too technical, depending on the individual perspective. Personal experiences and the perceived experiences of others were significant sources of knowledge and provided information about what it is like to become ill, what triggers exist to cause illness, and how one can manage the condition on a day-to-day basis.

### Living with an LTC

The core category identified in this study is *living with a long-term condition*. People make informed decisions in relation to their condition. They decide whether or not to seek medical advice when experiencing symptoms, using past experience as well as other information to aid the decision-making process. They choose whether to continue taking medication, and whether to adopt non-drug treatments. In addition, they decide whether to accept risks and consider their lifestyle behaviours.

Having learnt from previous experiences, those with thrombophilia who had had DVT or PE tended to look for the symptoms experienced when they had thrombosis. For example, one individual who had had both a DVT and a PE looked for the symptoms she had experienced before, leg pain and breathlessness, echoing the stories of other participants.

I do sometimes as I said get pains in my legs, but there's only been a couple of times where I've sort of convinced myself that I might have another clot … yeah and I I've never ever since been breathless like I, if I had another PE, boy would I know about it. (P111)

Treatment decisions—both taking medical advice and resisting it—were also informed by knowledge. For instance, one woman had made the decision to stop taking warfarin. Despite seven clots, she had experienced severe side effects from the medication. She was fully aware of the risks associated with her decision, but decided that these were outweighed by the benefits at that time.

I went off the Coumadin. I haven't had swelling erm, since then, I've increased my running, I haven't broken any bones … it's almost as if right now I'm going back to what I like … it bothers me because I know I'm playing with fire … Do I have a false sense of security because I have been off for six months and this is the longest I've gone without an incident. (P143)

Lifestyle decisions were also made by those with LTCs, often based on experience or knowledge gained in other ways. Those with asthma indicated that alcohol could aggravate their condition, which one woman managed by using her reliever inhaler.

As I've got older, I've found drinking alcohol brings on my asthma. Erm, I don't know why that is, just, and it's only sometimes, only some kinds of alcohol—white wine weirdly seems to bring it on which is odd. (P141)

Often individuals make decisions informed by knowledge and experience. Some of these are made on a daily basis, for example, taking medication. Others are made only at pertinent times, for example, by those with thrombophilia considering taking a long-haul flight. Individuals also make decisions which, though reversible, are intended to be permanent (e.g., to start or stop taking medication). These decisions depend on the circumstances at any specific time.

**Figure 1:**
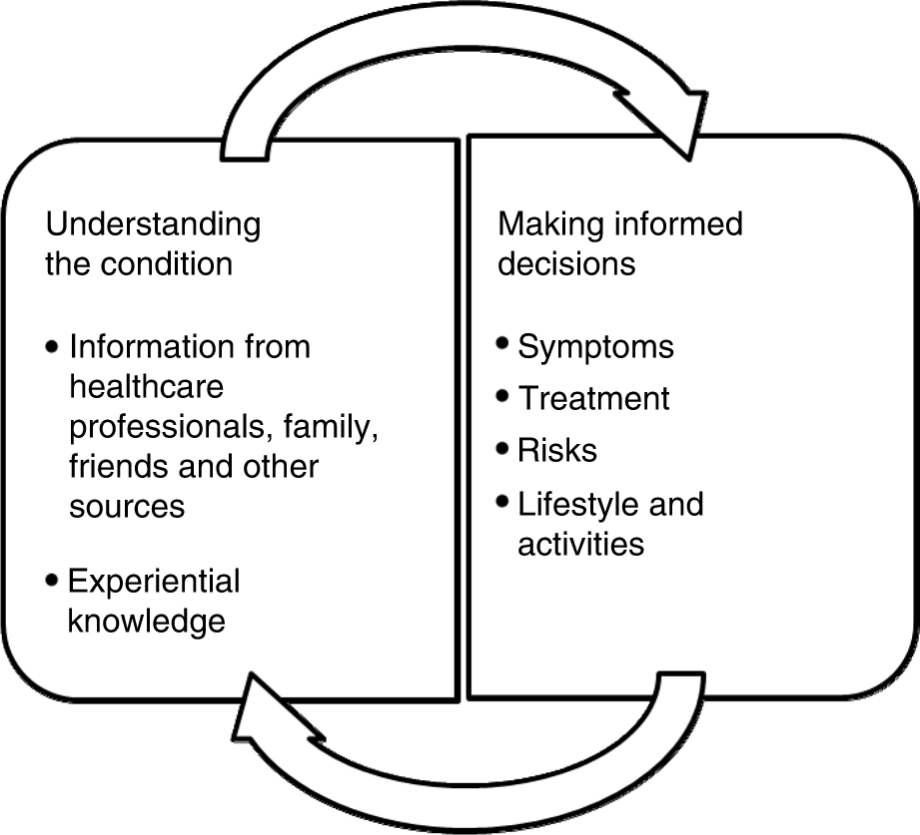
Diagrammatic representation of feedback loop existing between understanding and informed decisions made by those with a long-term condition.

By making decisions, individuals take responsibility for managing and a degree of ownership for their condition. A feedback loop exists which enables the outcomes of decisions to contribute to knowledge and thus to influence future choices. This is represented diagrammatically in Figure 1.

Some people demonstrated through their comments that they had accepted their condition. This included any uncertainty it created for them and its consequences and implications, including the need to accept responsibility for its management. For those who had experienced a delay in receiving a diagnosis and, particularly, those who had become ill as a result, the past may also need to be accepted. Some individuals do not accept their condition and all its nuances. However, a change in circumstances can cause them to move from acceptance to non-acceptance or vice versa, for example, having recently left military service, one woman had stopped accepting thrombophilia and was questioning both the condition and her approach to its management.

Some individuals accepted that they had been advised to take medication to reduce their risk of having a clot, and did so in the knowledge that this was a long-term recommendation and that their medication might have side effects. For example, one individual mentioned that she would prefer not to have to take warfarin—“I'm just waiting for them to invent something else, yeah anything please that won't eat me away inside” (P112).

Uncertainty featured strongly in the comments of those affected by thrombophilia, with some accepting that the circumstances in which a clot might occur were uncertain, and they could not be sure of the potential cause and timing, whilst others were unable to accept this. One individual, who had experienced a number of clots both personally and of family members, accepted uncertainty as an inherent part of the condition.

Statistically speaking the chances of my having another clot are only even now about 12% more than the general population … just because you have it doesn't mean to say you're gonna get a clot. It's just one of those horrible things that happens. (P111)

Similarly, past events were accepted by some participants. Several of those with thrombophilia had inherited the condition, with one saying “At least then I know it's not my fault and anything I've done, it's just the hand I've been dealt I guess” (P112).

However, some participants did not accept the uncertainty that results from having a condition such as thrombophilia or previous occurrences. One woman had experienced repeated missed diagnoses over a period of decades and was aware that she may have miscarried as a result of the untreated condition. She struggled to understand how the condition had been missed for so long despite her frequent symptoms and consultations with healthcare professionals—“It's just such a frustrating feeling, you know, and it's not about sort of blame or I'm gonna sue them and all the rest of it, but you just think, god you know, all those flipping opportunities” (P121).

Thus, acceptance of the condition, including its implications and consequences, responsibility for managing it, any uncertainty inherent in it, and any adverse events which may have happened, forms a pivotal point for individuals with an LTC. Those who are able to accept their condition, in all its guises, will be more likely to live with it. However, those who do not accept it will live alongside their condition. Despite their lack of acceptance, these individuals had taken some ownership of their condition; they accepted their diagnosis and were making decisions.

Living with their condition was possible for some of those interviewed, and this led to it becoming part of their identity. They were aware of it most when it became necessary. People might adopt a changed outlook on life, acquire professional roles relating to their condition, and place it in the context of other illnesses they may have or be aware of.

In contrast, other individuals lived alongside their condition, which they found difficult to accept. It caused them concern and restricted their activities whilst drawing their attention. For these people, the condition existed separately from the self and was not part of their identity.

## Discussion

Based on these findings, it is possible to elucidate a theoretical model of how individuals with an LTC might learn to live with it. This is presented as:

1. Information and knowledge, including that based on experiences, result in constructs which represent a condition as it exists for that individual.
2. Constructs are used as the basis on which individuals make decisions relating to a range of activities. A feedback loop offers further understanding which may reinforce or update existing constructs, or lead to the formation of new ones.
3. Individuals may accept their condition. Acceptance depends on how a person understands and conceptualizes it before diagnosis, at diagnosis, and post-diagnosis in an ongoing process.
4. Individuals who are knowledgeable about their condition, who are able to make informed decisions and who accept the condition may be described as living with the condition. For these people, the condition is part of their identity and integrated into their everyday lives. This may be contrasted with those for whom the LTC exists alongside their selves. For this group, the condition has not become part of the self and their everyday life, but is an unwelcome burden which interrupts their day-to-day life by causing concern and restriction.

**Figure 2:**
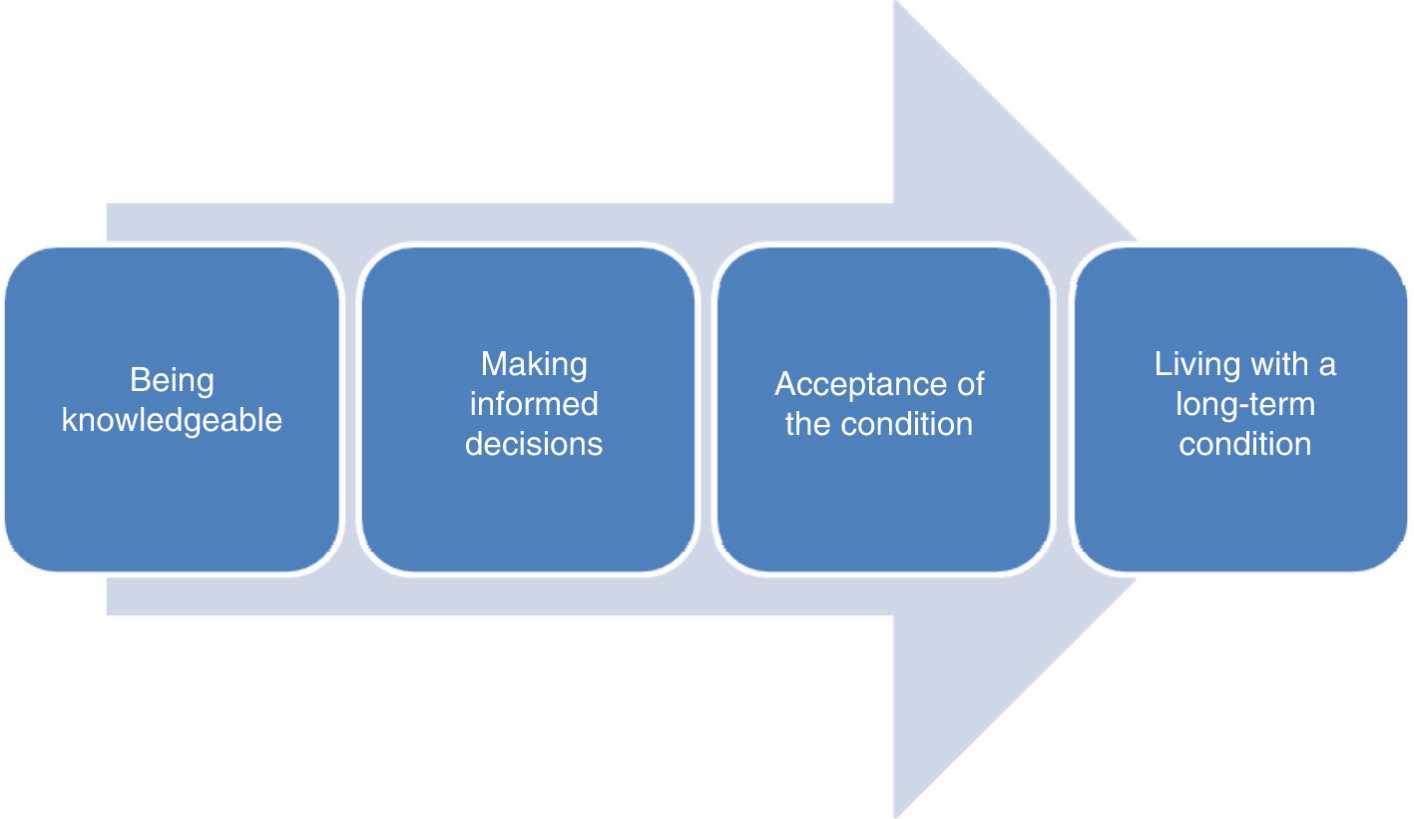
Diagrammatic representation of the factors which enable individuals to live with a long-term condition.

This theory is summarized in Figure 2.

With regard to how participants obtained information and its usefulness for their understanding of their condition, individuals obtained information from a range of sources. The findings reflect certain aspects of the concept developed by Edwards, Wood, Davies, and Edwards (2013) of ‘distributed health literacy’, which describes how people draw upon the health literacy skills of others with regard to health information. Those in the current research shared knowledge and understanding and used their social networks to access and evaluate information in a distributed way. Delays in diagnosis sometimes resulted from a delay in seeking medical advice, a factor which has also been seen in studies of those with rheumatoid arthritis (Townsend, Backman, Adam, & Li, 2013), cancer (Smith, Pope, & Botha, 2005), and heart attack (Finnegan et al., 2000; Horne, James, Petrie, Weinman, & Vincent, 2000). Alternatively, the diagnosis may be missed by the healthcare professional consulted; the literature demonstrates that thrombophilia is not alone in being misdiagnosed, with diabetes, stroke (Burgess, Cowie, & Gulliford, 2012), neurological conditions (Peters, Fitzpatrick, Doll, Playford, & Jenkinson, 2013), and back pain (Lillrank, 2003) being dismissed by professionals. The experiences of others are used to help make meaning of illness and support decision-making according to the findings of Ziebland and Herxheimer (2008).

Beliefs about one's condition were an important factor in the decisions individuals made. Choices with regard to medication confirm the findings of previous research, which demonstrates that people consider prescribed medication and choose to take them or not depending on what they consider to be the appropriate option (Atkin & Ahmad, 2001; Kopnina & Haafkens, 2010; McHorney & Gadkari, 2010; Pound et al., 2005; Teh et al., 2009). Similarly, decisions were made concerning activities based on one's beliefs about their potential impact on the condition. These were frequently based on experience rather than medical advice.

A range of other factors also affected the process experienced by those with the study conditions, including acceptance of any uncertainty caused by the condition, its consequences and implications, and prior events. The concept of acceptance is seen in the literature relating to LTCs and can be linked to mental well-being (McCracken, 1998; Viane et al., 2003), higher knowledge levels (Adams, Pill, & Kones, 2007), and reduced anxiety (Helder et al., 2002; Schüssler, 1992).

Concepts similar to that of living with a condition found in the literature include achieving harmony (Delmar et al., 2005), being ill and happy (Carel, 2007), quality of life (Drummond, 2000), having control over a condition (Snadden & Brown, 1992), and feeling well (Sanderson et al., 2010).

## Conclusion

This study, using a grounded theory methodology, provides a theoretical model of how individuals with an LTC can make sense of this, learn to adapt to it, and integrate it into their life. Whilst it used the examples of thrombophilia and asthma, the theoretical contribution made can be applied to other conditions which may exist in the background for much of the time, but can on occasion cause life-threatening illness. For many people, an LTC is part of who they are. The condition is recognized as conferring a risk, but is not considered to be especially serious for much of the time. Those affected are able to recognize times when they may be ill and are able to make appropriate decisions to medicate or seek treatment. They are also able to make other decisions, for instance about treatment, often without a great deal of effort because they are aware of the parameters of what they consider to be acceptable. They will return to consider the condition at pertinent times, for example, when needing to make different treatment decisions, but for much of the time it will exist in the background of their lives. Accepting the condition as part of their identity will greatly enhance their well-being.

Conversely, for other people, an LTC is a burden. It draws their attention, generates questions, and causes concern. They are unlikely to feel knowledgeable and do not accept all aspects of the condition. In particular, they struggle with the uncertainty inherent in having an LTC whose progression is not necessarily clear, with its consequences or with past events which they may struggle to come to terms with. The condition exists alongside these people and holds a negative meaning; this group is less likely to experience well-being.

The theory presented offers an explanation of how those with an LTC can learn to live with it, through the acquisition of knowledge, through making informed decisions, and through acceptance. Awareness of this might be useful for individuals with an LTC, who will be able to use it to identify the circumstances under which they are enabled to live with their condition and thus experience well-being. Health professionals will be able to recognize where they can offer help, advice, and support to aid patients in achieving well-being.
